# Understanding Autism in Schizophrenia

**DOI:** 10.1100/2012/254091

**Published:** 2012-04-26

**Authors:** Arnaldo Ballerini

**Affiliations:** Psychiatric Specialization School, University of Florence, Via Venezia 14, 50121 Florence, Italy

## Abstract

Detachment from external reality, distancing from others, closure into a sort of virtual hermitage, and prevalence of inner fantasies, are the descriptive aspects of autism. However, from an anthropological-phenomenological point of view, in schizophrenia, the autistic mode of life can arise from a person's being confronted with a pathological crisis in the obviousness of the intersubjective world, essentially a crisis in the intersubjective foundation of human presence. The “condition of possibility” of the autistic way of being is the deficiency of the operation that phenomenology call empathetic-intuitive constitution of the Other, an Other which is the naturalness of evidence of being a subject like me. The theme of the Other, of intersubjectivity, has become so central in the psychopathological analysis of schizophrenic disorders because the modifications of interhuman encounter cannot be seen as the secondary consequences of symptoms but constitute the fundamental disorder of schizophrenic alienation. Revision of the concept of autism from the original definition, centered on the prevalence of inner fantasies, leads to the profound change with the vision of autism as “loss” and “void.” I call attention to possibility of phenomenological research to understand autistic world starting from this “void.”

The image of retreat, of separation from the world that is *common* (the everyday world) and *in-common* (shared by all), has from the beginning been central to the concept of autism and has remained one of its most evident descriptive aspects.

However, rethinking the phenomenon of autism means asking ourselves what angles of study are adopted to view it, and how it disappears when viewed from other angles. The horizon on which the concept of autism arises most richly and meaningfully is undoubtedly that of phenomenological psychiatry. From a phenomenological point of view, in schizophrenia, the condition that can be called the autistic mode of life can arise from a person's being confronted with a pathological crisis in the obviousness of the intersubjectivity of the world, that is, a difficulty in the foundation of the Other in the mind of the subject.

The interdependence between constitution of the Self and constitution of the Other explains the pathological experiences that express at the same time the crisis in the relationship with oneself, with a Self that has become a stranger, and with the Self of others, which has lost the naturalness of being another subject like me, with his normal distance from me, and it is often perceived by the autistic person as an “enigma.” Really, the autistic person would like to have a personal “Theory of Mind” to understand others, “possibly in a file,” as a patient told me.

Daily life, which is normally a problematic in its silent foundation of the constitution of the Other, appears cracked in schizophrenic autism, and autism can be its perceivable expression, like an atmosphere enveloping this manner of being, even before a possible delusion arises. Autism is thus at its base a sort of “void,” which can nonetheless be grasped, and which has been explored, after Eugen Bleuler, in the schizoid personality and schizophrenia as a “loss of vital contact with reality” by E. Minkowski, as “inconsistency of natural experience” by L. Binswanger, and as “loss of natural evidence or overall crisis of “common sense” by W. Blankenburg—in my opinion all are broadly overlapping theses.

All this research touches on the source, the root, the condition of possibility for the manner of life we call autism to manifest, a manner which often creates anthropological figures revolving around “strangeness,” “oddity,” *Verschrobenheit*, as Binswanger calls it.

Autism is a concept that goes beyond psychiatric diagnoses, even if it finds in the sphere of schizophrenia its most complete and pervasive expression and characterization. It can thus be proposed that even if not all forms of autistic style are in themselves diagnosable as schizophrenic disorders, the core forms of the schizophrenias are unthinkable except as autistic. Indeed, for Minkowski, autism is not one of the symptoms of schizophrenia: it is schizophrenia itself, as a peculiar mode of existence. At the intersection of the cultural backgrounds deriving from Bergson's philosophy and from phenomenology, at the intersection of the concepts of “generative disorder” and of involvement of the entire personality, the turning point is Minkowski's conception of “autistic activity.” It is through this, the autistic person can no longer be considered as simply turned in on his fantasies, and autism is a phenomenon understood purely as interiorization, as “passive *rếverie*, absorption of the personality merely by its inner life” (E. Minkowski). The observation is that sufferers from schizophrenic autism are not all passively turned in on themselves, but even when they do act in the world, their activity has a profoundly morbid stamp to it, because they throw their act out into the world without taking account of it, in the sense of it not being sufficiently in agreement with “common sense,” so that the act seems “strange,” “inconsistent,” “stiff,” “overdone,” and burns out in itself (*actes sans lendemain*).

Minkowski wrote in *Au-delà du rationalisme morbide* [1] that “We feel, from time to time, the need to isolate ourselves from the environment and remain alone with ourselves … But … do we therefore try just the same to exclude thoroughly every outside influence? No, of course not. On the contrary, we let the environment act on us. In this way, while isolating ourselves, we remain in contact with the environment.” The author notes that in this relationship, always fluid and changing, between isolating ourselves to safeguard our own originality and receptiveness to the environment, they are not precepts of mental health, except maybe in the elastic fluidity of this relationship, whose “regulating element” is totally unrationalizable; Minkowski calls it *sentiment d'harmonie avec la vie.*

If, paraphrasing Pascal, “life has its reasons that reason has no way of explaining,” it from this prerational, preverbal harmony with the world of life is the sense of limits and measure derives: at bottom, the obvious evidence of the world as intersubjective.

On another level, we can say that the “co” that grounds being as cobeing appears in the autistic way of acting to be even more openly lacked than in inertia-isolation-withdrawal, which we can always imagine as the fruit of the disturbed person's immersion in a fantasy world, attributing to him a richness and exuberance that may be exaggerated, just as a New York lawyer did with his “scrivener” Bartleby, in Melville's story.

How ever, the clinical descriptions that have been made of the autistic condition in its different behavioral, relational, and affective aspects concern also the capacity of the “person” to react and to take a position, so to speak, with regard to more basic generative disorders. For example, the autistic breaking of relationship does not depend on a lack of nearness in the spatiality of the interpersonal world, but it is a reaction to a lack of distance, a defense against the risk of being absorbed by the world.

When dealing with autism, we must not hide the problems and difficulties involved, especially in the passage from the plane of phenomenological vision to the plane of clinical psychiatry. Autism, even before being a problem for clinical psychiatry, is a problem for the tradition of general psychopathology.

Indeed, above and beyond the analysis of individual pathological experiences, the concept of autism compels an anthropological attempt to move toward an approach to man as a whole.

Binswanger [2] writes that autism, from the anthropological perspective, means “subjugation of the Self by the World, worldification (*Verweltlichung*) or dismissal of the Self, de-ipseification”.

I emphasize the centrality in the anthropo-analytic discussion of autism, and in general of schizophrenia, of the concept of “subjugation by the world,” which represents the opposite of the free projection of oneself into the world, but is a process in which “…the accent of existence—as a priori being-in-the-world—is displaced from oneself to the world …” [3].

It is this crisis of the habitual rootedness in the intersubjectivity of human presence that transforms normal “being-with” into the unhappy defective autistic mode of being-with, a mode that does not infrequently go so far as to establish, in a sort of compensation for the basic deficient constitution of the Other, a human presence, even if in oppression and, at worst, in persecution.

However, autism is not, I repeat, in terms of an analysis of presence, just an unfortunate and deficient specification of being-with, but it contains also a defensive aspect, an attempt at resistance, even in the crisis of the ontological foundation of ipse-ness, to affirm oneself in any case in the empirical world. It is significant, as he has always reminded us, that psychopathology considers, concerning the theme of the restricted and oppressive spatiality of autism, that this lack of contact expresses the person's efforts to break free of the yoke of oppression placed on him by people and situations. Autism shows in its various empirical forms a defensive desire to be left in peace, which indicates just this loss of protective distance from the world.

Here, in my opinion, it is necessary to recall once again the distinction between the “condition of possibility” of the autistic way of being (the deficiency of the operation that phenomenology—from Husserl to Stein—call empathetic-intuitive constitution of the Other) and the ways in which the person manages, adapts, conforms, and even uses this same deficiency in his life.

In a text written in 1917, Husserl's glorious pupil Edith Stein identifies “empathy” as a basic problem “in that it is the experience of subjects other than ourselves and of their life experience (*Erleben*) … the phenomenon of a psychophysical individual who is clearly different from a physical “thing”” and presents itself “as a living sensible body that possesses an I, an I that perceives, feels, wants ….” It is in contact with otherness that self-ness “succeeds in differentiating itself from the otherness of the other,” Stein adds.

In an intersection of cross-references, empathetic movement thus means the constitution of the other as a person and the coconstitution of one's own Self.

The phenomenon of autism has been the guiding thread of the thought of numerous well-known scholars, but studying autism is an experience of exciting insights and resignation to disappointment, a concept that seduces the mind of the psychopathologist along the path to understanding the core of schizophrenia and repeatedly disappoints the clinician's aspiration to exactness and reliability.

This is where we pay the price of the generic nature of the formula “loss of contact with reality,” which while grasping a reality that is evident in psychoses, becomes simply a synonym for “psychosis.” So that, while not every autism is delusional, neither is every delusion autistic, in the sense described above.

Moreover, from an excursus on the forms that the concept of autism has taken in the history of ideas in psychiatry, one might draw the impression that more than autism, there exist various autisms, given that—as Glatzel [4] asserts—autistic traits are inherent in every psychopathological disturbance. This is certainly true, at least as regarding psychotic disorders, when one makes the altered relationship with reality, the all-encompassing viewpoint on autism, without examining the manners and pathways that structure this altered relationship with reality, and above all the “core” of autism as a difficulty in the empathetic constitution of the Other. So, as Tatossian emphasizes, we must not mistake an “I” that has simply withdrawn from the world, as in melancholy, for an autistic “I,” as in schizophrenia.

In recent years, the term autism has virtually disappeared from psychiatric diagnostic manuals about schizophrenia. Nonetheless, even if the word autism practically never occurs in today's psychiatric categorizations, its traces can be discerned—albeit degraded to behavioral shells, which tell us nothing about the subjectivity of the person—in the formulation of the negative symptoms of schizophrenia.

The opinion has been widespread, and today is returning, it seems to me, that considers autism to be a central element of schizophrenia. However, the autism we are thinking about today is more Minkowski's with his vision of autism as “loss” and “void” (“*autisme pauvre*”) than the autism of E. Bleuler's original definition, broadly centered on the prevalence of inner fantasies.

In effect, the history of the attempts to define the syndrome or syndromes of the group of schizophrenias seems to me largely a history of failures. No matter how many symptomological criteria we manage to bring together, it is the “way of being” peculiar to schizophrenia that typically colors the various symptoms.

No psychotic symptom is specific to schizophrenia (but, at most, some of them might be suspect) if it is not immersed in the autistic atmosphere.

When autism and its conditions of possibility come forth as a probable precursor of schizophrenia, we refer to situations of “imbalance” in which, for reasons connected both with intrapersonal and situational dynamics (at bottom, the separation between the I and the world is an *après coup* of reason), the style and way of living interwoven with autistic elements lose their internal norm; they do not hold any more, and the person is thrown off-balance by anguish. This is certainly not the fated outcome of the autistic profile which, as a characteristic, embraces all the “schizophrenic spectrum,” from personality disorders to psychosis.

Autism thus appears, on the pervasive level of absolutization of a single way of being, as the pathoplastic core of schizophrenia, which then takes expressive form in symptoms, of which retreat may be just one mode of behavior, and withdrawal into oneself may be a forced necessity and at the same time a means of defense. In other words, perhaps, the course of schizophrenia may be initiated when psychotic states of consciousness meet up with a person marked by this problem in the constitution of the Other, when the “pathology of the consciousness” (in H. Ey's sense) takes form through a particular autistic fragility of the person.

The theme of the Other, of intersubjectivity, has become so central in the psychopathological analysis of schizophrenic disorders because the modifications of interhuman encounter cannot be seen as the secondary consequences of symptoms but constitute the fundamental disorder of schizophrenic alienation.

“In effect, if there were no interweave of interpersonal relationships, there would be no schizophrenics,” Kimura peremptorily wrote [5].

Indeed, constitution of the I and constitution of the Other are like two sides of the same coin: their crisis is the epiphany (manifestation) of autism.

The constitution of the Other is truly the fundamental condition of possibility for the world to be intersubjective, the foundational event of any encounter and the building of any interpersonal community and social network.

Otherness finally, is not something added secondarily to ipse-ness, but is part of the constitutive function of it.

“The Other is not reduced, as is too facilely considered acquired knowledge, to the otherness of a Somebody Else” [6].

The Other of Husserl's pairing (*Paarung*) of two subject-bodies, two *Lieb*; the Other constituted in us by empathy, which defines, delimits, actualizes, cobuilds our ipse-ness; the Other encountered from our earliest relationships as we go forward in the world, up to the Other as the creator and witness with us of the roles of the social network and the culture of shared meanings.

The Other is the “secret sharer” of Conrad's novel, who is a part of us and with us is embarked on our ship of ipse-ness.

On the contrary, an identity marked by a shaky constitution of the Other corresponds to a shadowy, volatile identity, which imprints the trait of autism on all the syndromes of the spectrum of schizophrenia, from the schizotypal to the schizophrenic, precisely in that, from the perspective of genetic psychopathology, the fragility of the of transcending into the other and of constituting it as a subject is projected onto the difficulty of individuation of the Self. The whole context of life and identity in intersubjectivity thus risks difficulty in emerging and affirming itself.

With regard to the problem of “transcendental constitution” of intersubjectivity, I wonder if we live in intersubjectivity because we have fundamentally an experience of the world in common, or because in it we experience a common, shared world. It is the alteration of this circuit that is expressed in the painful inability to be with another, to put oneself in the place of another, to “feel” how others are, to live the obviousness of their being subjects like us, like us the source of thoughts, affections, needs, and desires.

I might add that psychiatry essentially deals with those modes of existence, tragically compelled and tragically not free, that do not manage or find it hard to constitute themselves in coexistence, in the world that they nonetheless design: all the way to that extreme manner of being, marked by isolation and evanescent communication, which we call autism.

Solitude and the evaporation of communication: these are the two most common behavioral aspects of the eclipse of intersubjectivity. In the history of philosophical thought, solitude moves between wisdom and madness, between the search for a higher form of communication and the impossibility of communication. This dilemma between “desire” and “impossibility” is true also for the contradictory facets that the phenomenon of autism offers to the eye.

In any case, we must ask ourselves if the possibility of suspending “being-with,” (*Mit-Dasein)*, “co-esse,” to at least a certain point, may not be a possibility, even a saving one, that still belongs to life.

As seems to happen with certain schizotypes who precipitate into the loss of the obviousness of the shared world appear actively to consent to this [7], transforming it into a value.

The concept then of “communication,” which recurs so often in the psychiatric jargon about autism, is certainly not easily defined, and from a certain standpoint all the phenomenology of interpersonal relations can be viewed in terms of communication.

In “an ontologically broad sense” communication, “in which the articulation of being together is constituted,” it is not just “the transfer of lived experiences from the inner core of one subject to the inner core of another,” Heidegger notes this in *Sein und Zeit* [8]. The loss of communication in psychosis is much more than the crisis of the transmission reception of a message. This loss can signal the crisis of intersubjectivity in the world of life, given that the observable alterations in interpersonal encounter, as I have already offered, cannot be derived from a secondary manifestation, an incidental and variable symptom, but belong, plausibly, to the core of schizophrenic alienation.

Gadamer [9] writes “Language does not belong to the sphere of I but of We.” A preliminary way of being that does not descend from nor belong to the free disposability of the individual: I could say, in heideggerian terms, that language is an expression of the sphere in which human presence finds itself “thrown” (*Geworfenheit*).

The communication crisis permeates, to be sure, the so-called “negative” area of psychosis, but it can well be the precondition of possibility, an initial and perhaps basal point of schizophrenic psychosis, a sort of “anticipatory deficiency” in Janzarik's sense [10], a region in which “the word has given up being recognized”: and for J. Lacan this was the essence of madness.

However, the solitude of autism that connotes schizophrenia is not univocally made up either of introversion of absorption into one's own fantasies, or of social retreat, or of closing off communication. All these modes, I repeat, can be manifestations of the style of life that is called schizophrenic autism, if its enactment presupposes a “void,” which phenomenological research has explored and indicated using expressions that refer to the loss of the foundation of existence in the obviousness of the presence of the Other.

We know what stimulating contributions to the investigation of the construction of the Self in intersubjectivity are coming to us today from neurophysiological research, in extraordinary harmony with Husserlian phenomenology that affirmed the primacy of intersubjectivity, of “entropathy”. Indeed, current neurophysiological research proposes a circuit that seems to reproduce Husserl's phenomenological construct of *Paarung*, the pairing of two bodies in action, of two *Leib*. “Mirror neurons substantiate a multimodal representation of relations between organism and organism” [11].

An early, famous apologue by Jaspers compared phenomenological research and somatological research in psychiatry to the adventure of two explorations that reach different shores of the same continent, which is so vast that the two researches never meet. Perhaps at this point, it is worth remembering the virtuous relation between the two spheres of research; as a title by J. Z. Sadler [12] says “Eidetic and Empirical Research: a Hermeneutic Complementarity.”

Neuroscientific research on mirror neurons and phenomenological investigation of the constitution of the other demonstrate precisely this type of “virtuous complementarity” that stimulates us to reassess Jasper's apologist and attenuates the risk that psychiatrists run of being the mountebanks of psychiatry, as Naudin and others hinted [13].

Thus, we have before us neuroscientific data that are in strong relation with phenomenological insights and appear to be the possible elementary, constitutive bases for the complex field of social relations and also of social identity and its confluence with the other aspects of personal identity.

It seems certain to me that in schizophrenia, insufficiency in the “intrasubjective” constitution of oneself corresponds to a problem in the “intersubjective” constitution of oneself.

In the phenomenological study of consciousness, the un-discussed reality of the world of life derives from this basic certainty, or “presumption,” (*Vertrauen*) that joins together the naturalness of the Self, the Other, and the World.

The point of departure is the possibility of “intentional consciousness” which from the beginning, as appears in phenomenological *epoché*, constitutes the Other as different from one among many objects, inasmuch as it is a subject that, like me, thinks and recognizes me. The constitution of the Other is truly the basic condition for the possibility of the world being intersubjective, the foundational event of any encounter. Otherness is thus not something added secondarily to subjectivity, but it is part of the constitutive function of it.

To conclude, autism means a form of presence, a being in the world, in servitude or annihilation that only apparently can be overturned into its opposite, omnipotence, but always expresses—as Cargnello reminds us [3]—the impossibility of being truly oneself…, because of a lack of the authentic opening toward the You that is *conditio sine qua non* for any possibility for authentic existence.
